# The Royal Naval Medical Service: What It Offers to the Newly Qualified Medical Man

**Published:** 1914-09-05

**Authors:** 


### THE ROYAL NAVAL MEDICAL SERVICE.

### What it Offers to the Newly Qualified Medical Man.

Not to everyone who is standing at the borders of professional life is it given to see the place of his settlement lying just beyond the colleges across a narrow tract of house appointments. Many will travel the devious ways of "locums" and assistantships before their eyes fall on the looked-for opening and they pass into the land of the brass-plate and the private practice. For such another path is possible, with here and there a turning towards the same goal or ending in a settlement in the land through which it runs. The path of the Royal Naval Medical Service may be followed up to the high position of surgeon-general, of rank equal to that of a rear-admiral or major-general, or even to the exalted post of medical director-general, while at the end of every fourth year of full-pay service there is the possibility of retirement with a gratuity that will go some way towards the purchase of a partnership or practice.

### Navy Service or Private Practice?

If the Naval Medical Service is regarded as only an entry into professional life and weighed against the lot of the "locum" or assistant, its suitability as a means to the end of a private practice may be determined on other grounds than those of individual preference. With many the settled home will weigh heavily against the ship's cabin or the hospital quarters. On the other hand, the Naval Service can show many attractions that will go far towards turning the scale. The naval surgeon's work is unharassed by the cares of securing or retaining patients; his time is not wasted nor his strength consumed in journeys; his income is paid regularly without the worries of collecting fees; his sleep will be disturbed only on his appointed nights of duty; in his holidays he can dismiss all cares lest he should have occasion to regret his absence. In how many private practices are his opportunities of operative surgery to be found? Afloat he may be far from hospitals or specialists to take his cases from his hands. Ability will not go unrecognised; and in the Service hospitals at Haslar, Plymouth, Chatham, Malta, Hong Kong, Bermuda, Portland, Haulbowline, the Cape, and Gibraltar skill in surgery will find the amplest scope. Certainly the private practice affords some experience that does not fall to the naval surgeon's lot. Midwifery he probably will not regret. With some branches of pathology he whose patients are drawn from a body of picked men will never have to deal; but against that may be set an experience of tropical diseases that in ordinary practice are rarely seen. For research and laboratory work there are abundant facilities; and even sea-time gives opportunities of observation and study that the busy practitioner on shore may often envy.

### How to Enter the Service.

The first step towards entry into the Royal Naval Medical Service will be to obtain from the Director-General, Medical Department, Admiralty, S.W., the necessary forms of application, and the candidate, if the Director-General has signified his approval and the Medical Board has passed him as physically fit, will be allowed to present himself for a four days' examination held in London twice in the year. The subjects are medicine, including medical pathology and therapeutics, and surgery, including surgical pathology and clinical surgery. The successful candidates become acting surgeons, and if they are ready to enter on their duties receive pay at the rate of 14s. a day. It will sometimes happen that a candidate is holding a hospital appointment or has one in immediate prospect, and as much as a year may be allowed him for holding the post. During this time he will not receive naval pay, nor may he count this time in the four years' service that entitles him to a gratuity on retirement, but in other respects his seniority dates from the passing of the entrance examination.

The acting surgeon enters on a two months' course at the Naval Medical School at Greenwich, where he studies tropical medicine, bacteriology, and clinical pathology, hygiene, and skiagraphy. This is followed by a four months' course at Haslar of naval hygiene, recruiting, physical training, and diving and submarine work. After passing an examination in these subjects he receives his commission as surgeon in the Royal Navy.

Meanwhile he will notice that the courses of instruction are carried out by naval medical officers, and that the Service, therefore, has posts to offer with extra pay to those who show peculiar aptitude for special studies and the work of instruction.

The surgeon's pay rises by a shilling a day every two years, till after the sixth year it reaches £328 10s. a year. He may retire at the end of the fourth year with a gratuity of £500. If he elects to continue in the Service, about the end of his sixth year he will enter on a five months' course at Greenwich. This course embraces the compulsory subjects of clinical medicine and clinical surgery, operative surgery, practical anæsthetics, ophthalmology, clinical pathology, and hygiene. Optional subjects are bacteriology, throat, nose, and ear diseases, and skiagraphy. Instruction is given partly at the Naval Medical School and partly at the *Dreadnought* Hospital. Arrangements are also made for courses at the civil hospitals in London.

### Experience, Prospects, and Pay.

No one need grow rusty in the Navy Medical Service. Ships are not always at sea. The majority of the fleet is concentrated in home waters, and at the naval hospitals at the three home ports voluntary courses of instruction are arranged, and every facility is given for laboratory work and the study and practice of bacteriology, clinical pathology, skiagraphy, and kindred subjects. After fourteen years' service an optional course of three months' duration can be taken at Greenwich, designed to afford senior officers the opportunity of refreshing their general knowledge of surgery and medicine and of making themselves familiar with recent advances. For this course there is no fixed syllabus, but individual requirements can be suited.

At the end of the eighth year of service the surgeon can retire with a gratuity of £1,000, or, if he has passed the examination that closes the six months' course at Greenwich and has served three years at sea, he is eligible for promotion to the rank of staff surgeon. Earlier promotion may be earned by distinguished service or conspicuous ability. The staff surgeon receives a pay of £1 a day, rising by successive increments to £456 5s. a year.

In comparing naval pay with the income of a practice on land it must not be forgotten that the naval medical officer has lodging provided for him, draws rations at sea, and receives a compensatory allowance in hospital, so that his living expenses are low. There are also extras in the form of charge pay for the senior medical officer of a ship and the medical officer in charge of a hospital or sick quarters, flag allowance on a flagship, etc.

At the end of the twelfth and sixteenth years of service there are again opportunities of retirement, with gratuities of £1,500 and £2,250 respectively, or the staff surgeon, after eight years' service in that rank, except in cases of special promotion, may proceed to the rank of fleet surgeon, a position which carries pay beginning at £492 15s., rising to £638 15s. a year, and entitles him after twenty years' service to a pension of £1 a day, which longer service will raise by degrees till on his compulsory retirement at the age of fifty-five it reaches the sum of £1 10s..

Service beyond the age of fifty-five is for those alone whose ability lifts them to the higher ranks. Professional ability in the Naval Medical Service is not limited to the skilful treatment of sick and wounded, but includes also a capacity for administrative and organising work. Not only may a medical officer have the supervision of all the details of hospital administration, but the principal medical officer of a fleet has a general responsibility for the sanitary conditions, and the state of the medical appliances in the various ships, and on him falls the duty of planning the transport of the wounded and advising the Commander-in-Chief. Talents for such services, in addition to professional skill, lead their possessor to the higher ranks of deputy surgeon-general and surgeon-general, to which promotion "will be made strictly by selection and will be confined to officers proved to be fitted both professionally and as administrators."

[*Further detailed particulars will be found on page* 636.]

